# Epidemiology of Infant Dengue Cases Illuminates Serotype-Specificity in the Interaction between Immunity and Disease, and Changes in Transmission Dynamics

**DOI:** 10.1371/journal.pntd.0004262

**Published:** 2015-12-11

**Authors:** Hannah Clapham, Derek A. T. Cummings, Ananda Nisalak, Siripen Kalayanarooj, Butsaya Thaisomboonsuk, Chonticha Klungthong, Stefan Fernandez, Anon Srikiatkhachorn, Louis R. Macareo, Justin Lessler, Julia Reiser, In-Kyu Yoon

**Affiliations:** 1 Department of Epidemiology, Johns Hopkins School of Public Health, Baltimore, Maryland, United States of America; 2 Department of Biology, University of Florida, Gainesville, Florida, United States of America; 3 Department of Virology, Armed Forces Research Institute of Medical Sciences, Bangkok, Thailand; 4 Queen Sirikit National Institute of Child Health, Bangkok, Thailand; 5 Division of Infectious Diseases and Immunology, Department of Medicine, University of Massachusetts Medical School, Worcester, Massachusetts, United States of America; 6 Dengue Vaccine Initiative, International Vaccine Institute, Seoul, Korea; University of North Carolina at Chapel Hill, UNITED STATES

## Abstract

**Background:**

Infants born to dengue immune mothers acquire maternal antibodies to dengue. These antibodies, though initially protective, decline during the first year of life to levels thought to be disease enhancing, before reaching undetectable levels. Infants have long been studied to understand the interaction between infection and disease on an individual level.

**Methods/Findings:**

Considering infants (cases <1 year old) as a unique group, we analyzed serotype specific dengue case data from patients admitted to a pediatric hospital in Bangkok, Thailand. We show differences in the propensity of serotypes to cause disease in individuals with dengue antibodies (infants and post-primary cases) and in individuals without dengue antibodies (primary cases). The mean age of infant cases differed among serotypes, consistent with previously observed differential waning of maternal antibody titers by serotype. We show that trends over time in epidemiology of infant cases are consistent with those observed in the whole population, and therefore with trends in the force of infection.

**Conclusions/Significance:**

Infants with dengue are informative about the interaction between antibody and the dengue serotypes, confirming that in this population DENV-2 and DENV-4 almost exclusively cause disease in the presence of dengue antibody despite infections occurring in others. We also observe differences between the serotypes in the mean age in infant cases, informative about the interaction between waning immunity and disease for the different serotypes in infants. In addition, we show that the mean age of infant cases over time is informative about transmission in the whole population. Therefore, ongoing surveillance for dengue in infants could provide useful insights into dengue epidemiology, particularly after the introduction of a dengue vaccine targeting adults and older children.

## Author Summary

Infants born to dengue immune mothers acquire maternal dengue antibodies. These antibodies, though initially protective, decline during the first year of life to levels thought to be disease enhancing, before reaching undetectable levels. We show that in this population, DENV-2 and DENV-4 almost exclusively cause disease in the presence of dengue antibody, despite infections occurring in others. We also observe serotype-specificity in the mean age of infant cases, consistent with differential waning of antibody to each serotype. These results highlight serotype-specificity in the way the immune response interacts with infection to cause disease. In addition, we show that the mean age of infant cases over time is informative about transmission in the whole population. Therefore, ongoing surveillance for dengue in infants could provide useful insights into dengue epidemiology, particularly after the introduction of a dengue vaccine targeting adults and older children.

## Introduction

DENV is a flavivirus that exists as four serotypes. Infection with one serotype leads to long-term immunity to that serotype. There is also a short-term period of cross-protection to other serotypes [1, 2] followed by an indeterminate period during which infection by another serotype may lead to more severe disease [3]. One theory for this increased severity is antibody dependent enhancement, whereby non-neutralizing antibodies bind to the virus and facilitate viral entry into cells and increased viral replication [4]. The overwhelming majority of hospitalized cases in regions where all four serotypes circulate are due to post-primary infections [5]. Infants born to dengue-immune mothers receive dengue antibodies, and, over the first year of life, experience an accelerated version of the susceptibility pattern that individuals experience during a lifetime in endemic areas: there is a short period of universal protection lasting a few months after birth, followed by a period also lasting a few months in which infections are more likely to be severe possibly through the action of antibody dependent enhancement [6].

Infant cases of dengue have been an important group for studying dengue immunopathogenesis. Previous studies have described the disease presentation and age distributions of infants in Thailand, Vietnam, Indonesia and the Philippines [7–9], as well as considering the interaction between antibody titres and disease [6, 10–12]. Infant cases may also be an important group for understanding other aspects of the epidemiology of dengue at population scales. There are two main advantages to evaluating infant cases for studying the interaction between immunity and disease. First, at a population scale and even at individual scales, infants have fairly uniform antibody titers across serotypes and, thus, eliminate the uncertainty of timing and nature of past exposures that exists when considering serotype differences in disease severity among older children. Second, the time period that infants are at high risk of infection with severe outcome is relatively short, thus providing information on forces of infection in the population at this time.

In the current study, we analyzed dengue case data from Queen Sirikit National Institute of Child Health (QSNICH) from 1973–2012 to investigate dengue in infants (cases <1 year old). We sought to elucidate intrinsic differences in the propensity for different DENV serotypes to cause disease among patients with pre-existing antibodies by examining serotype distributions in hospitalized infants, compared to other age and immunity groups. We also examined possible relationships between antibody levels and disease outcome by examining the age of severe cases among infants. Finally, we considered changes in dengue case numbers and mean age of infant cases over time and what these changes revealed about the force of infection (FOI) of dengue and population level transmission. This work is important for the study of dengue pathogenesis and epidemiology and is particularly relevant to the development of vaccines. An understanding of potentially protective antibody titers could inform vaccine immunogenicity targets and could clarify the interactions among serotype, immunity and disease outcome when interpreting population level vaccine trial results. Since no vaccine approaching licensure currently plans to target those under 1 year of age, this group could also be an important resource in Phase 4 studies as they will be easily identified as non-vaccinees, and can be used to characterize indirect effects of vaccine campaigns as well as characterize temporal patterns in population level transmission after the introduction of vaccines.

## Materials and Methods

### Study Design

The analyzed data were obtained from public health samples collected during passive surveillance of hospitalized dengue cases from 1973 to 2012 at QSNICH, a 420-bed tertiary care pediatric hospital located in Bangkok, Thailand, that serves as a Thailand Ministry of Public Health (MOPH) dengue referral center for Bangkok. Ninety-nine percent of the dengue cases were ≤15 years of age. Acute and convalescent blood samples from clinically suspected dengue inpatients at QSNICH were tested for evidence of DENV infection at the Armed Forces Research Institute of Medical Sciences (AFRIMS) laboratory in Bangkok. The case data up until 1999 have been presented previously [13] and an updated analysis is in submission (Nisalak *et al*., submitted to AJTMH). Techniques used for measuring antibody titers and detecting virus have changed over the years (see [13] Table 2). In brief, acute blood samples were tested by viral isolation and/or hemi-nested reverse transcriptase polymerase chain reaction (RT-PCR) as previously described [14–18]. Acute and convalescent blood samples were tested by dengue serological assays as previously described [19–22]. Primary infection refers to the first DENV infection in an individual and was determined serologically by dengue hemagglutination inhibition assay (HAI) and/or dengue IgM/IgG capture enzyme-linked immunosorbent assay (ELISA) according to published criteria [22]. Post-primary infection refers to any DENV infection subsequent to primary infection and was also determined serologically [22]. The retrieval and analysis of coded pre-existing data in this study was approved by the QSNICH and Walter Reed Army Institute of Research Institutional Review Boards. Blood samples from passive surveillance were originally collected at QSNICH for public health purposes. All data analyzed were anonymized.

### Statistical Analysis

For the purpose of this analysis, cases were grouped into three groups: 1) primary cases aged less than 1 year old, which we refer to as infant primary cases, 2) primary cases aged ≥1 year old which we refer to as non-infant primary cases, and 3) post-primary cases of all ages. Only 40 of 21,090 post-primary cases were <1 year of age; these cases were included in the post-primary group, but their inclusion in this or the infant primary group did not alter the results. For each group, we calculated the proportion of cases that were of each serotype, and for each serotype, the proportion of cases that were in each group. Using Pearson correlations, the correlation between the annual numbers of cases in each group for each serotype was assessed.

For infant primary cases, we calculated the mean age for cases of each serotype over all years and the mean age for all serotypes for each year. Using generalized linear models, we assessed trends over time and the relationship between annual mean age and annual proportion of all cases in infants. Analysis was performed using R version 3.0.2 (R Foundation for Statistical Computing, Vienna, Austria) [23].

## Results

**Fig 1:**
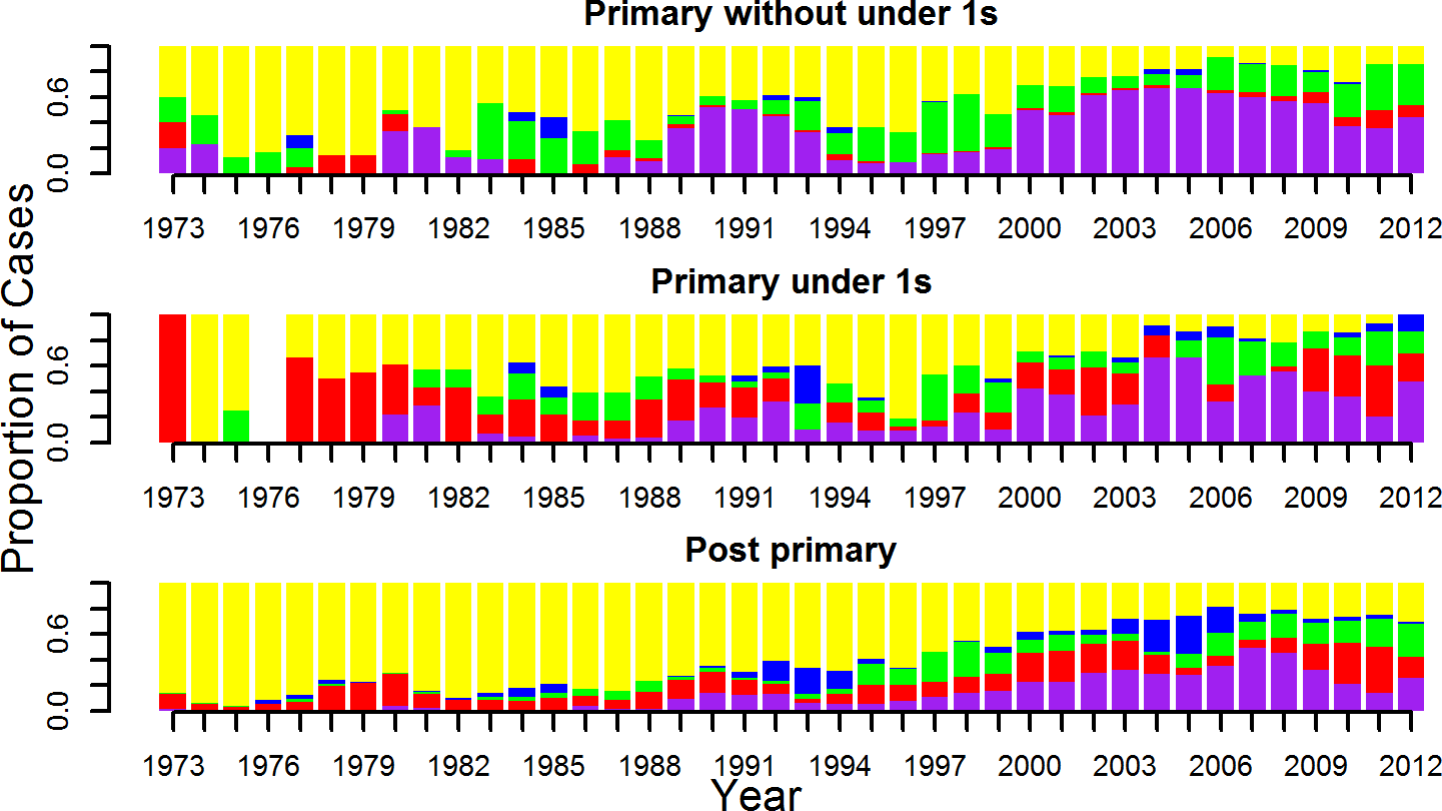
Figures show the serotype distributions of primary cases in 1) primary cases in non-infants, 2) infant cases and 3) post-primary cases. Serotypes DENV-1 to -4 are in purple, red, green and blue, respectively, untyped is in yellow.

**Fig 2:**
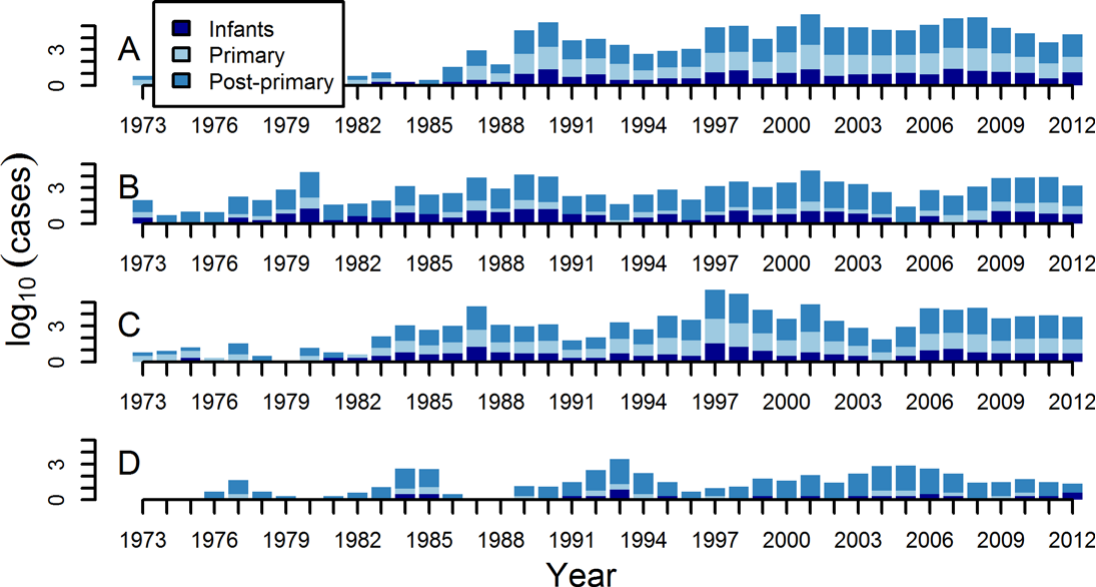
Each figure shows for each serotype individually (A-D: DENV-1 to -4) the number of primary cases in infants, the number of primary cases in non-infants, and the number of post-primary cases each year. Cases are shown on a log_10_ scale. Primary cases in infants are in dark blue, post-primary cases are in mid blue, and primary cases in non-infants are in light blue.

The serotype distribution in primary infant cases was more similar to the post-primary cases than to primary cases in non-infants (Table 1 and Fig 1). For both post-primary and infant primary cases, around 35% of cases were DENV-1 and and 31% of cases were DENV-2. This is significantly different to primary non-infant cases where a much greater 57% of cases were DENV-1 and only 5% were DENV-2. For DENV-3 there are slight, non-significant differences between post-primary and infant primary with 22% of post-primary and 27% of primary infant cases due to DENV-3, and these are both significantly less than the 37% of primary non-infant cases that were due to DENV-3. For DENV-4 there are significant differences across all 3 groups with 12% of post-primary cases, 4% of infant primary cases and only 1% of primary non-infant cases due to DENV-4 (Table 1 and Fig 1). Although disease was still more common in post-primary compared to primary cases for DENV-1 and DENV-3 (73% of cases were post-primary), primary infections with these two serotypes caused a substantial amount of disease in dengue naïve individuals. For DENV-2 and DENV-4 however, almost all of the cases of these serotypes were post-primary cases (92% and 96%, respectively). The percentage of cases that were untyped was the same for the primary infant and non-infant cases (both 40%), compared to post-primary cases (53%) (Table 2 and Fig 2).

**Table 1:** Proportion of serotyped cases in each group (primary in non-infants, primary in infants, and post-primary) for each serotype.

Group	DENV-1	DENV-2	DENV-3	DENV-4	Total
Primary in non- infants	0.57	0.05	0.37	0.01	1
	[0.55, 0.60]	[0.02, 0.07]	[0.34, 0.39]	[0, 0.04]	(1734)
	(990)	(83)	(639)	(22)	
Primary in Infants	0.37	0.32	0.27	0.04	1
	[0.34, 0.42]	[0.27, 0.36]	[0.23, 0.31]	[0, 0.09]	(632)
	(238)	(198)	(170)	(26)	
Post-primary	0.35	0.31	0.22	0.12	1
	[0.33, 0.36]	[0.30, 0.32]	[0.21, 0.24]	[0.11, 0.13]	(9717)
	(3356)	(3014)	(2210)	(1137)	

Table shows proportion and 95% multinomial confidence intervals in square brackets. Case numbers are in parentheses. The accompanying figure, with the data by year, is Fig 1.

**Table 2:** Proportion of cases of each serotype that are in each group (primary in non-infants, primary in infants, and post-primary).

Group	DENV-1	DENV-2	DENV-3	DENV-4	untyped
Primary in non-infants	0.22 (990)	0.02 (83)	0.21 (639)	0.02 (22)	0.09 (1151)
Primary in infants	0.05 (238)	0.06 (198)	0.06 (170)	0.02 (26)	0.03 (428)
Post-primary	0.73 (3356)	0.92 (3014)	0.73 (2210)	0.96 (1137)	0.88 (11373)
Total	1 (4584)	1 (3295)	1 (3019)	1 (1185)	1 (12952)

Table shows proportions of cases in each group that are of each serotype. Case numbers are in brackets. The accompanying figure, with the data by year, is Fig 2.

There were positive, significant correlations within each serotype each year between the number of infant primary cases, non-infant primary cases, and post-primary cases in each year, i.e., the number of primary non-infant cases of a serotype each year was associated with the number of infant primary cases and, separately, associated with the number of post-primary cases of that serotype each year (see Fig 2 and Table 3). For DENV-1 and DENV-3, the correlations between the numbers in each group were between 0.78 and 0.95, while for DENV-2 and DENV-4, these correlations were lower between 0.40 and 0.60.

**Table 3:** Correlations between the case numbers of each serotype in each immune group each year, p-values are shown in brackets.

	Correlations between annual case numbers of (p-values)		
Serotype	Infants and primary	Primary and post-primary	Infants and post-primary
1	0.84 (<0.001)	0.91 (<0.001)	0.78 (<0.001)
2	0.60 (<0.001)	0.51 (<0.001)	0.60 (<0.001)
3	0.87 (<0.001)	0.94 (<0.001)	0.79 (<0.001)
4	0.40 (<0.05)	0.46 (<0.005)	0.60 (<0.001)

**Fig 3:**
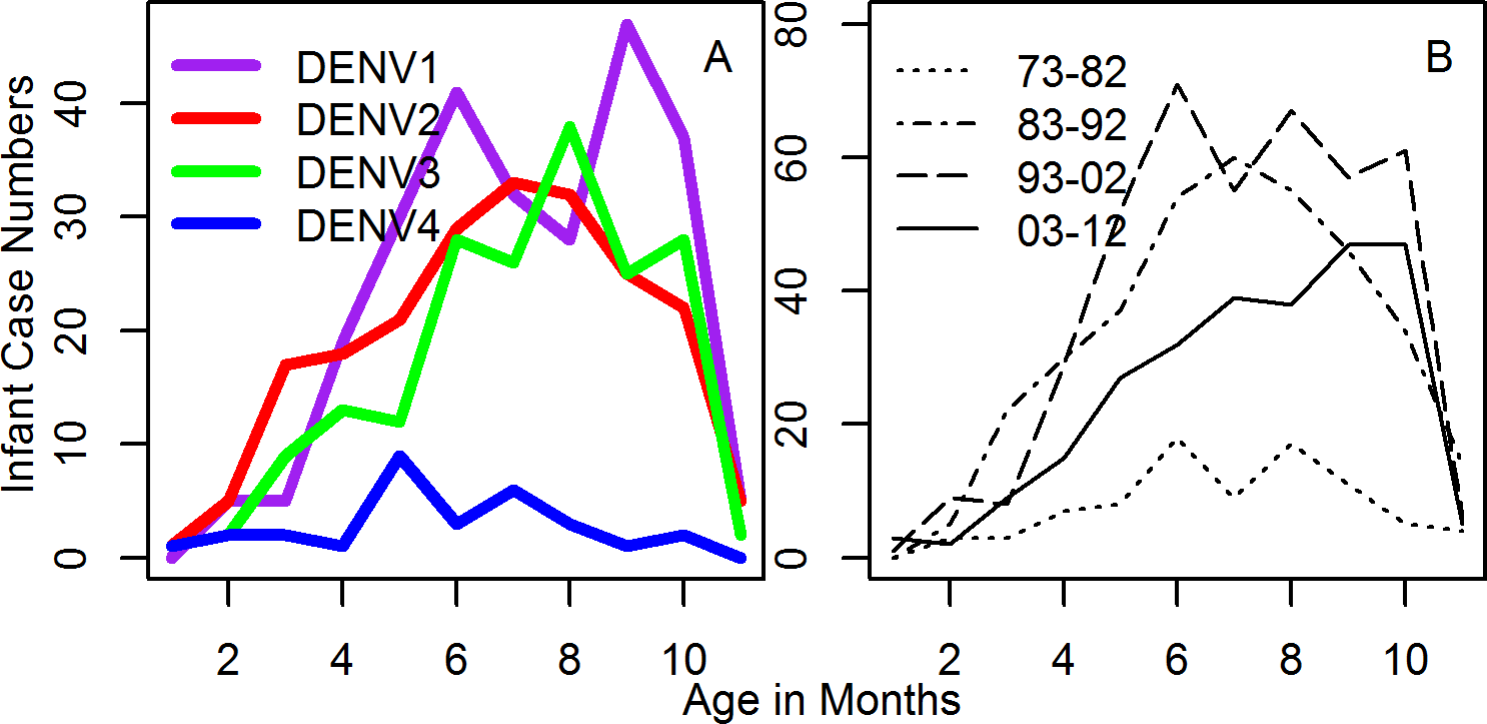
Figures show: A) number of cases in each month age group by serotype over all years, B) number of cases in each month age group by decade over all serotypes.

The mean age of all infant primary cases was just under 7 months (6.7 months [95% CI: 6.6, 6.9 months]). Age distributions by serotype are shown in Fig 3A. The mean age was highest for DENV-1 and DENV-3 (both 7.3 months [95% CI: 6.9, 7.5 months]), with DENV-2 slightly lower (6.7 months [95% CI: 6.5, 7 months]) and DENV-4 the lowest (5.7 months, though with the widest CIs [95% CI: 5, 6.5 months]).

**Fig 4:**
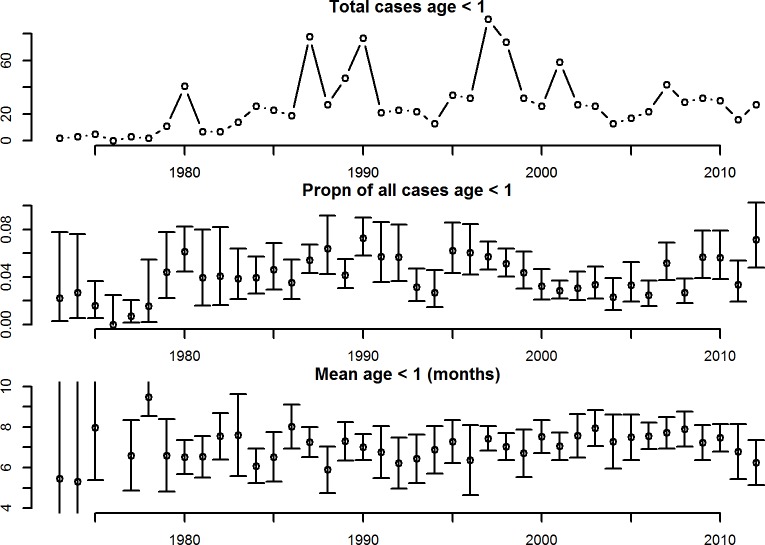
Figures show: 1) Absolute number of infant primary cases, 2) proportion of all cases that were in infants, and 3) the mean age of infant primary cases (in months).

Finally, we considered the trends over time in the mean age of infant primary cases and the infant primary cases as a proportion of all cases (Fig 4). There was a significant positive correlation between mean age and year (correlation 0.31, p-value: < 0.05). This increase in mean age was clear from 1990 to around 2007, but there was a suggestion of a decrease after 2007. Age distributions by decade are shown in Fig 3B. Using a linear model, there was a significant relationship between proportion of cases that were in infants and the mean age, year and the interaction between mean age and year (p-values all <0.01, coefficients: mean age: 18, year: 0.005 and mean age*year: -0.009). This relationship showed that during the years of increasing mean age of infant cases (1990–2007), there was a decrease in the proportion of all cases that were in infants; and during the years of decreasing mean age (after 2007), there was an increase in the proportion of cases that were in infants (Fig 4).

## Discussion

The serotype distributions of hospitalized dengue cases in different immune groups, as presented in this paper, add to the evidence that differences in the outcome of infection by each serotype depends on immune status. The paucity of primary cases of DENV-2 and DENV-4 has been shown in previous studies in Thailand [5]. The presence of infant primary cases with these serotypes, suggests that dengue naive individuals ≥1 year of age are exposed to these serotypes, but that these exposures do not result in hospitalized disease. There are two non-mutually exclusive ways to interpret these findings: (1) DENV-1 and DENV-3 were more likely to cause disease in non-immune individuals compared to DENV-2 and DENV-4, or (2) DENV-2 and DENV-4 were more likely to cause disease in an enhanced post-primary infection than DENV-1 and DENV-3. These results from the infants suggest that the former is the most likely explanation, with the correlations between the annual case numbers in each group (infant primary, primary non-infant and post-primary) being lower for DENV-2 and DENV-4, than for DENV-1 and DENV-3, suggesting the immune status of the population plays a larger role in the dynamics of DENV-2 and DENV-4. These differences may also explain previously seemingly contradictory results where a relationship was shown between infant DHF and increased levels of enhancing activity in sera for DENV2 [6], but not DENV3 [10]. In addition, there is a suggestion that DENV-4 is under represented in the infant primary cases compared to the secondary cases. This could be explained by a lower force of infection for DENV-4 (consistent with the fewer observed cases) leading to fewer exposures in this early time period. Differences between the serotypes in the propensity to cause disease in immune and non-immune individuals should be considered in the context of vaccination trial results with seemingly differential efficacy across serotypes, as with the Sanofi Phase 2b results [24]. Could the observed effect of the vaccination for DENV2 be because of the differential outcomes of DENV2 exposure in naïve and non-naïve individuals?

The mean age of infant primary cases was similar to previous studies [7, 8]. The observed differences in the mean age in infants by serotype (highest for DENV-1 and DENV-3 followed by DENV-2 then DENV-4) could be due to two non-mutually exclusive reasons. Firstly, the force of infection could have been higher for the serotypes with the lower mean ages (so infections occurred on average earlier). Secondly, differential waning of antibody titers by serotype could lead to potential enhancement occuring at different ages for the different serotypes. The first explanation would suggest a higher force of infection for DENV-2 and DENV-4 compared to DENV-1 and DENV-3. Previous work has suggested R_0_ and thus the FOI may be higher for DENV-2 and the considerable numbers of DENV-2 cases [25] would be consistent with a high FOI for DENV-2. However, DENV-4 had lower incidence overall and serological studies do not suggest that DENV-4 has a higher R_0_ than other serotypes [25]. For antibody waning, previous work by van Panhuis *et al*. [26] indicated the fastest antibody waning to be for DENV-4 (with a mean titer at 6 months of 17 [95% CI: 12, 25]). The next fastest waning serotype was DENV-2 (with a mean titre at 6 months of 25 (95% CI: 21, 31), followed by DENV-3 and DENV-1 (mean titer at 6 months of 35 (95% CI: 29, 43)). This order of antibody waning is consistent with the observed serotype-specific infant mean ages, though these results may be specific to the assays performed. There could, of course, also be differences between the serotypes in the antibody response required for protection or to lead to enhanced infections, as observed in older individuals in a study in Kamphaeng Phet, Thailand [27]. With a population such as Bangkok, we would expect broad antibody responses in maternal antibody, however further study of maternal over time and in multiple populations would be of great interest.

The small but significant increase in mean age of infant cases over time (particularly in the 1990s to 2007) is consistent with the increase in mean age of dengue seen in the general population in Thailand [28]. One of the leading hypotheses for this increase is a reduction in the force of infection [28]. Both the trends in mean age of infant primary cases and the relative proportion of all cases that were in infants, are consistent with a decrease in FOI during this period (as the FOI drops we would expect to see fewer cases in infants as the chance of being infected in this first year of life drops). It is interesting that after 2007, a decrease in the mean age of infant cases, and an increase in the proportion of cases that were in infants was observed, suggesting an increase in FOI in this period. This decrease in mean age was also observed in primary cases of older age groups (Nisalak *et al*., submitted to AJTMH). Further years of data will be needed to determine whether this a persistent trend or a transient fluctation. We show a change in the mean age of infants over time and by serotype, however the numbers of cases in each year are too small to determine the changes over time for each serotype.

Our study suggests that infants, in addition to being informative about immune-mediated pathogenesis, could act as a sentinel population for understanding population-level transmission. Vaccines currently in development are unlikely to be given to infants [29, 30], and therefore, this age group will still be largely susceptible to infection even after vaccine introduction. With this lack of change in susceptibility, surveillance of this population could help determine whether transmission or simply disease presentation has altered due to vaccination. Whether infant susceptibility changes due to vaccine-derived maternal immunity will also be an important question to address in follow up studies. One would hope that ultimately, infants will be protected by herd immunity, however, if protection is suboptimal for some serotypes, is greater against severe disease as opposed to just infection, or vaccine coverage is low, the force of infection could be maintained at close to pre-vaccination levels. Therefore, infants will still be at substantial risk for infection and be an important population to observe to understand immunity and transmission.

## Supporting Information

S1 Checklist STROBE Checklist. (DOC)
